# Evaluate the safety and efficacy of dura sealant patch in reducing cerebrospinal fluid leakage following elective cranial surgery (ENCASE II): study protocol for a randomized, two-arm, multicenter trial

**DOI:** 10.1186/s13063-022-06490-8

**Published:** 2022-07-20

**Authors:** Andrew P. Carlson, Emma M. H. Slot, Tristan P. C. van Doormaal, on behalf of the ENCASE II study group, E. H. J. Voormolen, J. W. Dankbaar, P. Depauw, B. Brouwers, M. R. Germans, E. Baert, J. Vandersteene, C. F. Freyschlag, J. Freyschlag, C. Thomé, F. Zenga, F. Penner, A. Abdulazim, M. Sabel, M. Rapp, T. Beez, M. Zuccarello, E. Sauvageau, K. Abdullah, B. Welch, D. Langer, J. Ellis, A. Dehdashti, J. VanGompel, B. Bendok, K. Chaichana, J. Liu, A. Dogan, M. K. Lim, M. G. Hayden

**Affiliations:** 1 Department of Neurosurgery, University of New Mexico, 1 UNM, Albuquerque, NM 87131 USA; 2 Department of Neurology and Neurosurgery, University Medical Center Utrecht, Utrecht, The Netherlands; 3 Department of Translational Neuroscience, University Medical Center Utrecht, Brain Center, Utrecht University, Utrecht, The Netherlands; 4 Department of Neurosurgery, Clinical Neuroscience Center, University Hospital Zurich, Zurich, Switzerland

**Keywords:** CSF leakage, Dura, Sealing, Prevention

## Abstract

**Background:**

Cerebrospinal fluid (CSF) leakage is a frequent and challenging complication in neurosurgery, especially in the posterior fossa, with a prevalence of 8%. It is associated with substantial morbidity and increased healthcare costs. A novel dural sealant patch (LIQOSEAL) was developed for watertight dural closure. The objective of this study is to clinically assess the safety and effectiveness of LIQOSEAL as a means of reducing intra- as well as postoperative CSF leakage in patients undergoing elective posterior fossa intradural surgery with a dural closure procedure compared to the best currently available dural sealants.

**Methods:**

We will conduct a two-arm, randomized controlled, multicenter study with a 90-day follow-up. A total of 228 patients will be enrolled in 19 sites, of which 114 will receive LIQOSEAL and 114 an FDA-approved PEG sealant. The composite primary endpoint is defined as intraoperative CSF leakage at PEEP 20 cm H_2_O, percutaneous CSF leakage within 90 days of, wound infection within 90 days of or pseudomeningocele of more than 20cc on MRI or requiring intervention. We hypothesize that the primary endpoint will not be reached by more than 10 patients (9%) in the investigational arm, which will demonstrate non-inferiority of LIQOSEAL compared to control.

**Discussion:**

This trial will evaluate whether LIQOSEAL is non-inferior to control as a means of reducing CSF leakage and safety

**Trial registration:**

ClinicalTrials.gov NCT04086550. Registered on 11 September 2019

**Supplementary Information:**

The online version contains supplementary material available at 10.1186/s13063-022-06490-8.

## Administrative information

Note: the numbers in curly brackets in this protocol refer to SPIRIT checklist item numbers. The order of the items has been modified to group similar items (see http://www.equator-network.org/reporting-guidelines/spirit-2013-statement-defining-standard-protocol-items-for-clinical-trials/).

**Table:** 

Title {1}	Evaluate the Safety and Efficacy of Dura Sealant Patch in Reducing Cerebrospinal Fluid Leakage Following Elective Cranial Surgery (ENCASE II): study protocol for a randomized, two-arm, multicenter trial
Trial registration {2a and 2b}.	Clinicaltrials.gov, NCT04086550
Protocol version {3}	Protocol version 2.0, February 2021
Funding {4}	Polyganics BV Rozenburglaan 15A 9727 DL Groningen, The Netherlands
Author details {5a}	^1^ Department of Neurosurgery, University of New Mexico, Albuquerque, NM, United States of America ^2^ Department of Neurology and Neurosurgery, University Medical Center Utrecht, Utrecht, The Netherlands ^3^ Department of Translational Neuroscience, University Medical Center Utrecht, Brain Center, Utrecht University, Utrecht, The Netherlands ^4^ Department of Neurosurgery, Clinical Neuroscience Center, University Hospital Zurich, Zurich, Switzerland
Name and contact information for the trial sponsor {5b}	Polyganics BV Rozenburglaan 15A 9727 DL Groningen, The Netherlands
Role of sponsor {5c}	Sponsor co-designed the study with the authors. The sponsor, who is funding the study, is involved in site selection and day-to-day performance of the trial with regards to device accountability and study training. The regulatory submissions, data monitoring and data analysis is performed by a contract research organization (CRO). Interpretation of the data is performed in accordance with the coordinating investigators. The sponsor integrates the information provided by the CRO and coordinating investigators into the study report, which is reviewed by the CRO and coordinating investigators for final approval of the report. The authors have full freedom in writing and submitting the academic report. Draft material should be provided to the sponsor for review at least 30 days prior to submission or presentation date. The sponsor may require that the Investigators delete from their documents any reference to the sponsor’s confidential information.

## Introduction

### Background and rationale {6a}

Cerebrospinal fluid (CSF) leakage is a frequent and challenging complication in neurosurgery, with a prevalence of 8% [1]. Risk factors include posterior fossa surgery, the size of the durotomy, and patient-related factors such as immune-status [2]. It is associated with substantial morbidity and increased healthcare costs, estimated at $10,000–15,000 per patient per leakage [3]. CSF-related complications include delayed wound healing, meningitis and surgical site infection which often require prolonged hospital stay, antibiotic treatment, reoperation, or external lumbar drainage. To prevent CSF leakage, various dural sealants were developed to augment watertight closure of the dura. Thus far, their use has not shown a significant effect in reducing the number of complications [1].

**Fig. 1:**
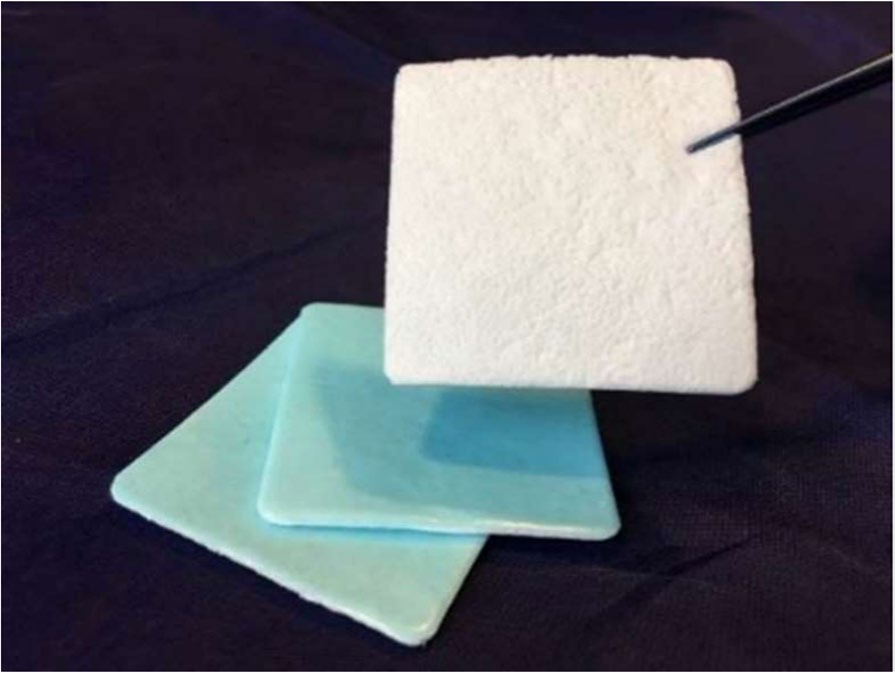
LIQOSEAL, investigational device. Length 8 cm, width 8 cm, and weight 1600 to 2000 mg

The sponsor of this study has developed a dural sealant patch (LIQOSEAL®, Polyganics BV) (Fig. 1). LIQOSEAL is designed to serve as an adjunct to primary dural closure in cranial surgery. Preclinical studies have shown that LIQOSEAL has advantages in dural adherence and burst pressure compared to other sealants [4]. The first in-human study [5] (ENCASE) showed that LIQOSEAL is safe and easy to use. However, a clinical comparative study testing its efficacy compared to control in humans has not been performed yet.

### Objectives {7}

The objective of the current ENCASE II study is to clinically assess the safety and effectiveness of LIQOSEAL as a means of reducing CSF leakage in patients undergoing elective posterior fossa intradural surgery, by showing non-inferiority compared to a control group.

### Trial design {8}

This study protocol adheres to the SPIRIT reporting guidelines. The SPIRIT checklist is supplemented as supplementary file 1. This study follows a randomized controlled, international, multicenter design with a 90-day follow-up. Patients will be randomized to receive LIQOSEAL or control (DuraSeal® (Integra) or Adherus® (HyperBranch, Medical Technology)) to be applied after the primary closure of the dura with suturing. Patients will be allocated in a 1:1 ratio to interventional device or control. The framework of this trial is noninferiority.

## Methods: participants, interventions, and outcomes

### Study setting {9}

This study will be conducted at up to 20 high volume neurosurgical centers in the USA and Europe. A complete list of participating sites can be found in appendix I.

### Eligibility criteria {10}

Preoperative inclusion criteria for participants

- Patients who are able to provide written informed consent prior to participating in the clinical investigation.
- Age ≥ 22 years.
- Patients who are able to comply with study requirements.
- Patients scheduled for elective surgery including a trepanation to reach the subdural infratentorial space (with lower limit of incision defined as the lower edge of C2) with closure of the dura.
- Female patients of child bearing potential must agree to use contraception from the time of signing the informed consent form (ICF) until 90 days post-surgery.

Preoperative exclusion criteria for participants

- Female patients who are pregnant or breastfeeding.
- Assumed impaired coagulation due to medication or otherwise.
- Presence of infection.
- Any type of dural diseases in planned dural closure area.
- Patients requiring re-opening of planned surgical area within 90 days after surgery.
- Known allergy to any of the components of LIQOSEAL, DuraSeal, or Adherus.
- Patients who previously received a LIQOSEAL, DuraSeal, or Adherus.
- Patients who previously participated in this study or any investigational drug or device study within 30 days of screening.
- Presence of hydrocephalus (which will not be resolved by the surgical procedure).
- Patients with contra-indication to MRI.

Intraoperative inclusion criteria for participants

- Surgical wound classification Class I/Clean.
- Minimally 5 mm of dural space surrounding dural opening.

Intraoperative exclusion criteria for participants

- Patients in whom elevation of PEEP has a potential detrimental effect.
- Patients who will require a CSF drain, electrodes, or other devices passing the dural layer or extra- to intracranial bypass surgery.
- Primary closure of the dura mater with material other than autologous material excluding fat.
- Patients in whom no intraoperative CSF leakage is present after primary closure of the dura mater with elevation of PEEP.
- A gap of > 3 mm after primary closure of the dura mater.
- Dural opening cannot be covered by LIQOSEAL (8×8 cm) with a 5-mm overlap.

Eligibility criteria for surgeons performing the intervention

Only neurosurgeons and neurosurgical residents trained for the protocol and application of both the interventional and control device will perform the study interventions. Virtual training will be provided by the sponsor during the site initiation visit and online revision material is available throughout the study. Only trained and signed off surgeons can perform study actions. A delegate from the sponsor also attends the first surgery if wished for by the local center.

### Who will take informed consent? {26a}

Informed consent will be collected by the principal investigator or designated study team member.

### Additional consent provisions for collection and use of participant data and biological specimens {26b}

Participants will be asked to consent or not to the use of their data for other research related to the investigational device, to be contacted for future studies and to share video/photographs of the surgery with the sponsor of the study.

### Interventions

#### Explanation for the choice of comparators {6b}

##### Investigational device description

The bioresorbable LIQOSEAL is indicated for use as an adjunct to standard methods of dural closure, such as suturing, to provide a watertight closure of the dura. LIQOSEAL consists of two layers: a watertight blue layer comprising biodegradable polyesterurethane and an adhesive white layer comprising biodegradable poly(DL-lactide-co-ε-caprolactone) copolymer and multiarmed N-hydroxylsuccinimide functionalized polyethylene glycol. This layer reacts with amines in the dural tissue in a moist environment, forming covalent bonds between the device and the tissue.

##### Control device description

The control arm of this study consists of two Food and Drug Administration (FDA) approved dural sealants for cranial use: DuraSeal and Adherus, indicated for use as an adjunct to standard methods of dural repair. Both consist of 2 components that when mixed together form an absorbable hydrogel. These products can be considered the current standard of care for dura sealing [6, 7].

#### Intervention description {11a}

1. The patient is electronically randomized for LIQOSEAL or control device on the day of surgery by trained personnel other than the operating surgeon.
2. The assigned dural sealant is taken out of the freezer/storage and placed in a non-transparent box.
3. First PEEP test (for safety).
4. Dura mater is closed with the standard method of suturing.
5. Hemostasis should be achieved.
6. The dura mater surface is rinsed from particles with physiological saline and kept moist.
7. Second PEEP test (CSF leakage confirmed).
8. Surgeon is unblinded and the dural sealant is applied.

- For LIQOSEAL: the dry patch is cut into the required size and positioned with the white side against the sutured area of the dura mater and compressed for 2 min with a moist gauze.
- For Duraseal and Adherus: the hydrogels are applied aiming at the sutured area of the dura mater, holding the device 2–4 cm away, until a thin coating (1–2 mm) is formed.

9. Third PEEP test (2 min after device implantation).
10. Standard closure of cranial defect and soft tissue will then be undertaken per surgeon standard.

#### Criteria for discontinuing or modifying allocated interventions {11b}

Once the patient is allocated to the control or interventional arm the patient can still be excluded from the study in case the intraoperative eligibility criteria are not met or in case the first PEEP test was not considered safe for the patient. Once the allocated intervention has been applied, this can be modified in case the patient reaches the primary outcome during the surgery (intraoperative CSF leakage at the third PEEP test). In such case, the neurosurgeon can undertake any actions deemed necessary to ensure optimal patient care in the situation.

#### Strategies to improve adherence to interventions {11c}

There is no patient action required to adhere to the intervention protocol. Surgeons are blinded until after primary closure of the dura to optimize adherence to the interventions independent of the allocated intervention.

#### Relevant concomitant care permitted or prohibited during the trial {11d}

Participants are not allowed to participate in any other investigational drug or device study. All other forms of treatment are permitted.

#### Provisions for post-trial care {30}

Insurance is in place, to enable compensation in the event of an injury to a participant.

#### Outcomes {12}

##### Primary endpoint

The primary endpoint is a composite endpoint defined as the occurrence of any of the following within 90 days of surgery:

- Wound infection defined in accordance with the Centers for Disease Control and Prevention guidelines as deep incisional (cat II) or organ or space infection (cat III) [8].
- Intraoperative CSF leakage after device application at a positive end expiratory pressure (PEEP) of 20 cm H_2_O.
- Percutaneous CSF leakage confirmed by β-2 transferrin test.
- Pseudomeningocele requiring puncture, external lumbar drainage or surgical re-intervention.
- Pseudomeningocele >20 cc as confirmed on MRI.

##### Secondary endpoints

*Safety*

- Device-related adverse events (AEs) and serious adverse events (SAEs) throughout the study up to 90 days after surgery.
- Complications requiring surgical re-intervention up to 90 days after surgery.

*Performance*

- Any pseudomeningocele as confirmed on magnetic resonance imaging (MRI) at day 90.
- Volume of pseudomeningocele as determined on MRI at day 90.
- Ease of use and application of the LIQOSEAL

#### Participant timeline {13}

**Fig. 2:**
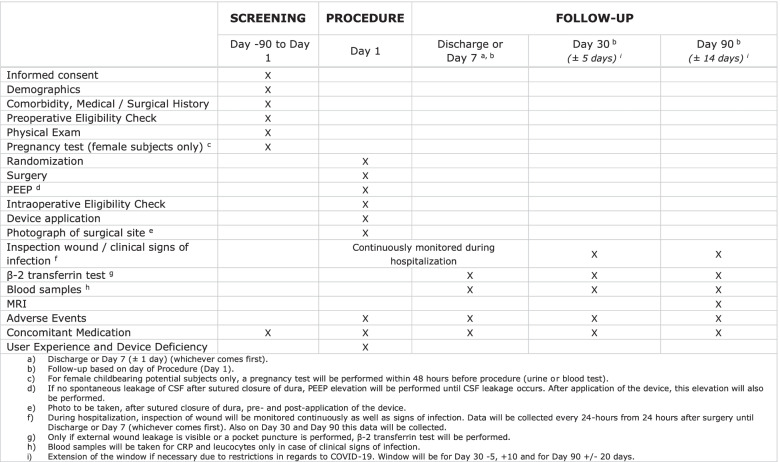
Spirit figure: schedule of enrolment, interventions, and assessments

**Fig. 3:**
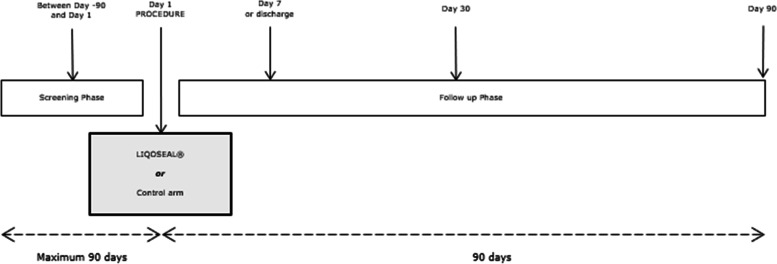
Study scheme

Screening will take place between day 90 and day 1 prior to surgery (Figs. 2 and 3). Follow-up will take place at day 7 or discharge (whichever comes first), day 30, and day 90.

During hospitalization, the patient will be monitored daily for clinical signs of infection and CSF leakage or swelling at the surgical wound. All patients will undergo an MRI on day 90.

#### Sample size {14}

“Success” was defined as absence of the composite endpoint. The success rate of the investigational product was set similar to that of the control devices combined. The combined success rate of the control devices was determined at 91%, based upon a comparative study of the two devices [6]. The non-inferiority margin was set at 10%, this was accordance with the design used by the control devices [6] and believed to be clinically acceptable. The power of the study is 80%, with a one-sided significance level of 5%. The expected rate of attrition is 10%. Under these assumptions, a total of 228 patients are required, 114 per arm.

#### Recruitment {15}

Prior to the start of the trial, estimations of recruitment numbers were provided by all participating sites. Recruitment is currently ongoing. Due to the impact of the covid-19 crisis, the recruitment time has been extended to 12 months.

### Assignment of interventions: allocation

#### Sequence generation {16a}

The randomization schedule will be generated using an Internet-based computerized randomization program within the research management platform (RMP) and electronic data capturing system Staicy v2.33 (IQVIA MedTech, Danbury, USA). The RMP is fully validated and 21 CFR part 11 (and EU Annex 11) compliant. It is developed under an ISO27001 certified quality management system.

Patients will be stratified by study site and type of cranial surgery (craniotomy or craniectomy) in blocks of 4 and randomized (1:1) at the start of surgery.

#### Concealment mechanism {16b}

Concealment of the randomization scheme is ensured by use of the RMP which shows the user the assigned randomization per individual patient for their site only.

The assigned device will be stored in a non-transparent box directly after randomization.

#### Implementation {16c}

Randomization is performed by trained personnel other than the operating surgeon by logging into the RMP to perform randomization for each individual patient at the time.

### Assignment of interventions: blinding

#### Who will be blinded {17a}

The surgeon is blinded for group allocation until finalization of the primary closure of the dura, by concealing the allocated device in a non-transparent box.

#### Procedure for unblinding if needed {17b}

The surgeon is unblinded after finalization of the primary closure of the dura, to apply the allocated device.

### Data collection and management

#### Plans for assessment and collection of outcomes {18a}

##### Preoperative data

Demographic information (i.e., gender, childbearing potential, age, length, weight, and body mass index), medical and surgical history (i.e., indication for surgery, allergies, tobacco use, medication use), and comorbidity of the patient will be collected. A physical exam is performed during screening. All female patients of child-bearing age will undergo a pregnancy test.

##### Intraoperative data

The device used and its size, LOT number, size of trepanation, any use of autologous material, and type of suture are recorded.

To determine the intraoperative CSF leak before and after the application of the device, the PEEP will be increased to 20 cmH2O for 20 s. First, this test will be performed before closure of the dura to determine safety for the postoperative intracranial field (control of hemorrhage, swelling or other potential adverse effects). Upon completion of the primary sutured dural closure and before the application of the sealant, the closure of the dura will be evaluated for CSF leakage by repeating the test. The patient is excluded if there is no leakage at PEEP of 20 cmH2O for 20 s. Two minutes after application of the device, the test will be performed for a third time to evaluate CSF leakage. All 3 PEEP elevations and application of the device will be recorded on video. A photograph will be taken before and after device application.

After the procedure, end users (surgeons, scrub nurses) will be invited to complete several closed-end questions regarding their user experience with LIQOSEAL.

##### Postoperative data

During the hospitalization, the subject will be monitored daily for clinical signs of infection. The surgical wound will be inspected daily starting 24 h after surgery. Blood analysis and a wound culture will be performed if there are clinical signs of infection. In case of CSF leakage from the incision, a β-2 transferrin test will be performed. Data will be collected every 24 h from 24 h after surgery until discharge or day 7 (whichever comes first).

The clinical data to be collected on the e-CRF includes the following: body temperature.

In case of signs of infection, the following data will be collected as well: C-reactive protein (CRP), leucocytes, and culture of wound.

The signs of infection will be classified to the Centers for Disease Control and Prevention (CDC) standard of Surgical Site Infection (SSI) and recorded in the e-CRF.

All subjects will undergo an MRI on day 90. The MRI will be performed to collect data on the presence and amount of pseudomeningocele (any subcutaneous fluid on T2) as well as the long-term thickness of dura mater and investigational device.

Independent radiologists will analyze the MRIs of all subjects for the outcome measurements. Each MRI will be evaluated by 3 independent radiologists, whereas the analysis then will be based on minimally 2 out of 3 evaluations (whom are in consensus).

Postoperative assessments are not blinded.

#### Plans to promote participant retention and complete follow-up {18b}

Participants will be contacted through telephone; after 3 unsuccessful attempts to reach the subject, a registered mail will be sent to the subject to indicate the need for a follow-up appointment. If these communications are unsuccessful, the subject will be considered lost to follow-up.

Data of withdrawn subjects, collected up until the point of withdrawal, will be preserved and used in the applicable analyses. The reasons for withdrawal will be compared between treatment arms, to assess potential bias in the analysis. The reason for discontinuation must be recorded in the source documentation and the e-CRF. Possible reasons for discontinuation of participation may include, but are not limited to, the following reasons:

- Subject decides to withdraw from the study
- Adverse events
- Lost to follow-up

#### Data management {19}

An electronic data capturing (EDC) system will be used to collect data on a secure, Internet-based electronic case report form (e-CRF), and image transfer software. The principal investigator (PI) or his/her designee at the clinical site will perform primary data collection by entering the data into the e-CRF. Only the PI or other predesignated personnel will be authorized to enter data using their unique login credentials. Each user access to the system will be tracked so that all data operations can be monitored and verified.

The e-CRF will be completed on a continuous basis starting from the point of enrollment to final follow-up.

A critical quality control shall be performed for the first 2 subjects by the sponsor’s designated data management team and queries issued where needed. Such queries must be reviewed by the monitor prior to alerting the site personnel to answer them.

After the monitor has done the source document verification and obtained satisfactory answers to eventual queries from the site, a full quality control shall be performed on the monitored data throughout the clinical investigation by the designated data management team and queries issued where needed. This process will be repeated till the end of the clinical investigation so as to allow for a timeline freezing of the data base for statistical analysis.

#### Confidentiality {27}

The investigator must ensure that subjects’ anonymity will be maintained. On e-CRFs or other documents submitted to the sponsor, subjects should not be identified by their names, but by the subject number. The investigator must keep a subject identification code list showing the enrolment number, the subject’s name, date of birth, and address or any other locally accepted identifiers. All information to be sent to the sponsor concerning patients and their participation in the study will be considered confidential. All data will be processed without identifiable reference to the individual patient.

#### Plans for collection, laboratory evaluation, and storage of biological specimens for genetic or molecular analysis in this trial/future use {33}

The collection and evaluation of laboratory tests will be performed according to the standard procedures per study site. The following laboratory tests may be applicable to participants in this this trial: pregnancy test, CRP, leucocytes, and β-2 transferrin test.

### Statistical methods

#### Statistical methods for primary and secondary outcomes {20a}

The primary analysis set will consist of a modified intention to treat analysis (mITT). Modified intent-to-treat (mITT) analysis set is a subset of ITT analysis set and will consist of all enrolled subjects (subjects who have signed the ICF, meet all inclusion criteria, meet none of the exclusion criteria and the investigational/control device has contacted the subject’s dura) with evaluable data for the primary endpoint.

The primary endpoint will be evaluated for statistical significance based on the Wald method for difference of proportions. If non-inferiority is met a one-sided significance level of 5%, the difference in success rates will also be evaluated for superiority at a one-sided significance level of 5%.

The primary endpoint will also be summarized by investigational site and cranial procedure type. Interactions of treatment and site/procedure type may be examined graphically or using logistic regression with success rate at day 90 as the response variable, and the study center (or procedure type), treatment group, and study center (or procedure type) by treatment group interaction as predictor variables in the model. If the results of the test show evidence of heterogeneity across sites/procedure types (i.e., *p*-value<0.15), then the treatment/procedure type effect will be evaluated further to identify any confounding factors.

All safety analyses will be based on subjects in the intention-to-treat (ITT) analysis set. The ITT analysis set will consist of all enrolled subjects (subjects who have signed the ICF, meet all inclusion criteria, meet none of the exclusion criteria, the investigational/control device has contacted the subject’s dura). Results based on the ITT analysis set will be analyzed according to each subject’s randomization assignment.

#### Interim analyses {21b}

There will be a safety stop after enrolment of 50 patients in the USA per the FDA. The 90-day safety data of the first 30 patients will be provided to the FDA to request approval to complete enrolment.

#### Methods for additional analyses (e.g., subgroup analyses) {20b}

As a supplementary analysis, the primary endpoint will be evaluated in a per protocol analysis (excluding subjects with protocol deviations) and ITT analysis. No supplementary of sensitivity analyses are planned for the safety and secondary endpoints.

#### Methods in analysis to handle protocol non-adherence and any statistical methods to handle missing data {20c}

By definition, the primary analysis of primary endpoint will be based on available data only, and no imputation for missing data will be performed. The supplementary analysis of primary endpoint based on the ITT analysis set will likely include some subjects who are excluded from the mITT analysis set and do not have evaluable data for the primary endpoint. Subjects who die prior to reaching the 90-day primary endpoint without experiencing any of the primary endpoint outcomes will be excluded from analysis. Data for any other subjects that do not have evaluable data at day 90 will be imputed using multiple imputation in the ITT analysis. The analysis of secondary endpoints will be based on available data only, with no imputation for missing data. Subjects who die or withdraw from the study for other reasons prior to experiencing a specific adverse event will be included in the denominator and assumed to have not experienced the event.

#### Plans to give access to the full protocol, participant-level data, and statistical code {31c}

The full protocol will not be published. An abbreviated protocol has been registered at ClinicalTrials.gov (number NCT04086550) prior to study initiation. The participant-level dataset and statistical code will not be granted for public access.

### Oversight and monitoring

#### Composition of the coordinating center and trial steering committee {5d}

There are two coordinating investigators for this trial: one from the USA and one from Europe. The study will be managed by a project manager and study coordinator employed by the sponsor. The sponsor’ study team, a PhD student, and both coordinating investigators will meet at a weekly basis. Day-to-day performance of the trial at the sites will be performed by site personnel trained for the study. Contact with the sites between sponsor, coordinating investigators, and sites’ study personnel will be at a regular basis, dependent on the need for the site based upon enrolment rate and site proficiency. The day-to-day performance of the trial by the sites will be supported and monitored by an independent CRO, who will be in contact with the sponsor, coordinating investigators, and sites’ study staff at a regular basis based on the need for the site based upon enrolment rate and site proficiency. An independent data monitoring committee (DMC) will be appointed consisting of at least 3 specialists in the field of neurosurgery, who will assess patients’ safety and trial progress based upon enrolment rate and (serious) adverse events on a regular basis.

#### Composition of the data monitoring committee, its role, and reporting structure {21a}

The data monitoring committee (DMC), consisting of at least 3 specialists in the field of neurosurgery, will review data relating to safety and performance and to ensure the continued scientific validity and merit of the study. Further details regarding the DMC can be found in its charter (Supplementary Material 2). Following each meeting, a formal report will be prepared, which will be sent to the sponsor after approval of all members of the DMC. The DMC is independent from the sponsor, and members have no competing interests.

#### Adverse event reporting and harms {22}

During the study, (serious) adverse events and (serious) anticipated and unexpected adverse device effects will be recorded; reporting will be done from point of enrolment till end of study.

The (principal) investigators shall report all adverse events and device deficiencies in the appropriate sections of the e-CRF and provide where requested by the sponsor, the necessary clinical or technical information that may contribute to clarifying the circumstances.

The (principal) investigators shall report all serious adverse events (SAEs) and device deficiencies (DDs) that might have led to a SAE if (a) suitable action had not been taken or (b) intervention had not been made or (c) if circumstances had been less fortunate, and new findings/updates in relation to already reported events to the sponsor and record in the e-CRF within 24 h after awareness of the event.

Any other reportable events as described above or a new finding/update to a reported event shall be reported immediately, but not later than 7 calendar days following the date of awareness by the sponsor of the new reportable event or of new information in relation with an already reported event.

#### Frequency and plans for auditing trial conduct {23}

Sponsor monitoring standards require full verification for the presence of informed consent, adherence to the inclusion/exclusion criteria, documentation of SAEs, and the recording of the main safety and performance endpoints. Additional checks of the consistency of the source data with the e-CRFs are performed according to the study-specific monitoring plan. An initiation visit will be performed before the first subject is enrolled. The first one site monitoring visit will take place within 21 calendar days of the first patient randomized. Further visits will take place at least twice a year. A risk-based monitoring approach will be utilized and the data points that are source data verified as well as the frequency of monitoring visits will be based upon enrollment, data integrity, and site compliance

#### Plans for communicating important protocol amendments to relevant parties (e.g., trial participants, ethical committees) {25}

No changes in the clinical investigation procedures shall be effected without mutual agreement of the principal investigator and the sponsor. The agreement of the changes must be documented by signing the corresponding clinical investigation plan amendments. All changes require notification to the EC/IRB and the Competent Authority/FDA (when appropriate).

### Dissemination plans {31a}

Within 1 year after the end of the study, a final study report with the results of the study, including any publications/abstracts of the study, will be submitted to the local ethics committee/institutional review board and the applicable competent authorities. Furthermore, the results of the study will be published in a scientific publication. If requested, the results of the study will be shared with participants.

## Discussion

This is the first randomized controlled trial in which the safety and efficacy of LIQOSEAL will be compared to the best current practice. This trial will evaluate whether LIQOSEAL is non-inferior to control as a means of reducing CSF leakage and safety.

### Trial status

Recruiting. First patient enrolled on 20 May 2021.

### Supplementary Information

**Additional file 1: ** SPIRIT checklist.

**Additional file 2: ** DMC Charter.

## Data Availability

The final dataset and the resulting study report will be included in the Trial Master File for the study. This will be archived at the Sponsor and the participating sites. Only dedicated personnel at the Sponsor and the participating sites will have access to the files. The files will be requested to be archived for 15 years after study conclusion.

## Appendix

List of participating sites

## Abbreviations

CSF: Cerebrospinal fluid
e-CRF: Electronic case report form
EDC: Electronic data capturing
ICF: Informed consent form
ITT: Intention to treat
mITT: Modified intention to treat
PEEP: Positive end expiratory pressure
PI: Principal investigator
RMP: Research management platform
