# Supplementary material for: Evaluate the safety and efficacy of dura sealant patch in reducing cerebrospinal fluid leakage following elective cranial surgery (ENCASE II): study protocol for a randomized, two-arm, multicenter trial
Source: Trials. 2022 Jul 20;23:581. doi: 10.1186/s13063-022-06490-8 (PMC9297260; doi:10.1186/s13063-022-06490-8)
Supplement: Supplementary file 2 — Additional file 2: DMC Charter. [file 13063_2022_6490_MOESM2_ESM.pdf]

Polyganics
Rozenburglaan 15A
9727 DL Groningen
The Netherlands

ENCASE II: Randomized, two-arm, multicenter study to evaluate the safety and efficacy of Dura Sealant Patch in reducing CSF leakage following elective cranial surgery

CONFIDENTIAL

Charter

Version: 1.0 **Date:** 24-MAR-2021

Revision history

Version	Date (dd-MMM-YYYY)	Changes
1.0	24-MAR-2021	Initial release

Note: This Charter will serve as the Standard Operating Procedure (SOP) for the Data Safety and Monitoring Board (DSMB).

Signature Page

I have read this charter and agree with its content. I will conduct my responsibilities on the DSMB as outlined herein.

Ruben Dammers, MD
DSMB Chairperson

April 9, 2021
Date Signature

William Peter Vandertop, MD
DSMB Member

Date Signature

Ricky Rasschaert, MD
DSMB Member

April 11, 2021
Date Signature

Prepared by: Ildephonse Ngabonziza, Safety Manager
IQVIA MedTech

Date Signature

Signature Page

I have read this charter and agree with its content. I will conduct my responsibilities on the DSMB as outlined herein.

Ruben Dammers, MD
DSMB Chairperson

Date   Signature

William Peter Vandertop, MD
DSMB Member

W.P.Vandertop Digitaal ondertekend door W.P.Vandertop Datum: 2021.04.07 12:31:14 +02'00'

Date   Signature

Ricky Rasschaert, MD
DSMB Member

Date   Signature

Prepared by: Ildephonse Ngabonziza, Safety Manager
IQVIA MedTech

DocuSigned by Ngabonziza Ildephonse

Ngabonziza Ildephonse

I have reviewed this document
13-Apr-2021 | 10:58:29 EDT

518AA3307B1C483C9CAD590BA7F68BA4

Date   Signature

TABLE OF CONTENTS

1 Abbreviations 4
2 Quality assurance 4
3 General 4
3.1 Introduction 4
3.2 Roles and responsibilities 4
3.3 Documentation 6
3.4 Membership 6
4 DSMB review procedures 6
4.1 Meetings 6
4.2 Data review 8
4.3 Safety updates 8
4.4 Stopping rules 9
4.5 DSMB Recommendations 9
4.6 Response to DSMB Recommendations 9
5 Amendments to the DSMB Charter 10
6 Archiving of DSMB Activities and Related Documents 10
7 Confidentiality 10
ATTACHMENT 1 - CONTACT DETAILS 11
ATTACHMENT 2 - DSMB MONTHLY updates 12
ATTACHMENT 3 - DSMB MEETING REPORT 13

1 ABBREVIATIONS

AE	Adverse Events
CA	Competent Authority
DSMB	Data Safety and Monitoring Board
EC	Ethics Committee
FDA	Food and Drug Administration
IRB	Institutional Review Board
SAE	Serious Adverse Events
SOP	Standard Operating Procedure

2 QUALITY ASSURANCE

The following reference documents have been used as the guidance for the composition and operation of the DSMB, as described in this Charter.

Reference	Title
ISO14155	Clinical investigation of medical devices for human subjects – Good clinical practice
FDA	Guidance for Clinical Study Sponsors: On the Establishment and Operation of Clinical Study Data Monitoring Committees.
EMEA/CHMP/EWP/587 2/03 Corr	Guideline on Data Monitoring Committees
Protocol	CIP-2<br>Randomized, two-arm, multicenter study to evaluate the safety and efficacy of Dura Sealant Patch in reducing CSF leakage following elective cranial surgery [ENCASE II]

3 GENERAL

3.1 Introduction

This Charter will outline the roles and responsibilities of the Data Safety and Monitoring Board DSMB established for ENCASE II study sponsored by Polyganics BV (hereafter referred to as Polyganics) and will serve as the Standard Operating Procedure (SOP) for the DSMB.

The charter defines the DSMB, its membership, and the purpose and frequency of its meetings. The charter also provides the procedures for ensuring confidentiality, the guidelines for communication, and an outline of the content of the reports provided to the DSMB.

3.2 Roles and responsibilities

Data Safety and Monitoring Board

The DSMB is responsible for assessing data during the course of a study in a manner that contributes to the scientific and ethical integrity of the study. The DSMB's recommendations will provide the sponsor with an overall scientific, safety, and ethical appreciation of the study, and should assist the sponsor in maintaining the rigor of the study design, with appropriate attention paid to the protection of human subjects.

The DSMB is responsible for determining its operational procedures and acting in accordance with its approved DSMB Charter. If changes to the Charter are required, amendments will be prepared by IQVIA MedTech and agreed to by the sponsor and DSMB.

The DSMB members will:

- Work according to the last approved version of the protocol and the procedures described in this charter;
- Review any protocol amendments, addendums or modifications since previous meeting;
- Define DSMB processes and study stopping guidelines prior to the first data review;
- Periodically review and monitor aggregated and individual subject data related to safety, data integrity, scientific validity and overall conduct of the study, to ensure the rights, safety, and welfare of the study participants;
- Monitor subject accrual and retention;
- Review formal interim safety analysis and evaluate the benefit/risk balance (if applicable);
- Provide in writing recommendations to Polyganics concerning the continuation, modification, or termination of the study;

In addition to the above, the DSMB Chairperson will:

- Serve in a leadership role and conduct DSMB meetings;
- Oversee the overall scientific integrity of data review;
- Review and approve the meeting report.

<u>IQVIA MedTech</u>

In addition to formation, operational support, and serving as a liaison between DSMB and Polyganics, IQVIA MedTech is responsible for overall coordination of DSMB activities to ensure that the DSMB maintains smooth and efficient operations throughout the study. IQVIA MedTech will:

- Compile and report (S)AEs to the DSMB, as appropriate;
- Compile and report overviews describing the progress of the study to the DSMB, as appropriate;
- Prepare relevant summary data reports in response to DSMB inquiries (This may include analyses not otherwise outlined in this charter, based upon findings);
- Facilitate the DSMB meetings;
- Maintain documentation and records of all activities;

<u>Polyganics</u>

The sponsor will be responsible for (but not limited to):

- Selection and formation of the DSMB;
- Communication with Competent Authorities (CA), Ethics Committees (EC) and investigators, in a manner that maintains integrity of the data, as necessary (This communication is not the responsibility of the DSMB);
- Promptly report potential safety concern(s) to the DSMB;

3.3 Documentation

Throughout the duration of the study, the DSMB will be apprised of all new safety information relevant to the device and the study. This includes providing the DSMB with a copy of the protocol in advance of the first meeting, as well as promptly providing any revisions. All safety reports issued by the sponsor and other pertinent documents relating to the study will be provided.

3.4 Membership

The DSMB consists of at least 3 specialists in the field of neurosurgery, with specialization and relevant clinical expertise documented by a recent signed and dated CV. The names of the DSMB members, their affiliations, and contact information are listed in ATTACHMENT 1 - CONTACT DETAILS.

The DSMB members are independent from Polyganics and the participating investigators without any financial, scientific, or other conflict of interest in the trial. Written documentation specifying the absence of any conflict of interest will be present prior to the start of the activities and will be collected by the sponsor. The absence of any conflict of interest will be verified during the first DSMB meeting. The members must immediately report any changes in the conflict of interest/ financial disclosure that occur during the course of the study. Any questions or concerns that arise regarding conflicts of interest will be addressed by the DSMB Chairperson with input from other DSMB members, IQVIA MedTech and Polyganics as necessary.

The DSMB members will receive financial compensation for their time and their expenses will be reimbursed. Details regarding financial compensation and payment schedule will be handled on an individual basis with each DSMB member and will not be described in this charter.

Members must have sufficient availability to attend all planned and ad hoc meetings as needed and must understand the time commitment required for data review for the duration of the trial.

The sponsor will appoint the DSMB Chairperson.

4 DSMB REVIEW PROCEDURES

4.1 Meetings

Frequency of meetings

The first meeting will be organized prior to the first patient enrollment for the members to:

- Form an understanding of the protocol and the definitions being used;
- Establish a meeting/data review schedule;
- Establish list of events that will trigger the DSMB review;
- Form the study modification and/or termination guidelines;

The DSMB will then meet according to the following schedule:

- Meeting 1: DSMB kick-off meeting
- Meeting 2: When the first 30 patients have been discharged from hospital or have reached 7 days post-operative, in case discharge is after 7 days post-operative)
- Meeting 3: When the first 30 patients accomplish the 30 days follow-up visit
- Meeting 4: When the first 30 patients accomplish the 90 days follow-up visit

- Meeting 5: to review the interim analysis report after 30 patients have completed the study (90-days follow-up)
- Meeting 6: When all patients have completed the study (90-days follow-up)

Of note, during the second DSMB meeting, the DSMB will review the meetings schedule and modify it as needed.

An emergency meeting of the DSMB may be called at any time by the Chairperson or any other party involved in the Study, should questions arise related to the patient's safety.

Polyganics and IQVIA MedTech Safety Team may seek the DSMB advice for any reason outside of scheduled meetings.

Meeting structure

Meetings may be convened as conference calls, as well as in person.

Meetings must be attended by at least 2 DSMB members. If 2 members provide different opinion, a 3rd member must be consulted.

If the DSMB decides to issue a recommendation to terminate the Study, all DSMB members present must vote for or against the recommendation. Majority vote will rule in the event of a split vote and a statement written by the committee members who did not vote with the majority, outlining their opinion (a minority report), should be appended.

An IQVIA MedTech facilitator will attend the DSMB meetings as a non-voting member in order to facilitate data presentation.

Minutes will be recorded by IQVIA MedTech, approved (via email) by the DSMB Chairperson and maintained by IQVIA MedTech in accordance with applicable statutory regulation. A copy of the minutes will be provided to the sponsor.

Each DSMB meeting can consist of an open session and a closed session. The open session may be attended by representatives of Polyganics. Only the DSMB members will have voting rights.

Minutes of the open session will be recorded by IQVIA MedTech. Minutes will be finalized upon signature of the DSMB Chairperson and maintained by IQVIA MedTech in accordance with applicable statutory regulation. A copy of the minutes will be provided to Polyganics.

The closed session will be restricted to the DSMB members. The IQVIA MedTech facilitator, who is not the member of the clinical (CPM/CRA) team, may attend the closed session upon DSMB approval. The minutes of the closed session will be recorded by the DSMB Chairperson or IQVIA MedTech facilitator if present. Minutes from the closed session will be recorded separately from the minutes of the open session and stored securely by IQVIA MedTech. Closed session minutes, finalized by signature of the DSMB Chairperson, will be maintained in confidence and retained until the end of study, after which the minutes will be provided to Polyganics.

4.2 Data review

IQVIA MedTech will provide suggested formats or templates to the DSMB for data presentation. The DSMB must review and approve the data elements presented. At subsequent meetings, additions or modifications to session reports may be directed by the DSMB on a one-time or continuing basis.

IQVIA MedTech will prepare and distribute data reports to the DSMB at least one week prior to the meeting.

The data presented in the reports must reflect both the need for the most complete information possible on study results and the need to ensure reliability and accuracy of the information included. The intent is to only provide source-verified (monitored) data to the DSMB; however, unmonitored data may be presented as well.

Reports for meetings of the DSMB consist of two separate parts: An Open Session Report and a Closed Session Report. Open Session Reports are distributed to DSMB members, sponsor staff, and other appropriate persons at least one week prior to a scheduled meeting. Closed Session Reports are distributed on the same schedule, but are only distributed to DSMB members, DSMB facilitator(s) and others as designated by the DSMB Chairperson.

The DSMB Chair will notify IQVIA MedTech if additional information required.

4.3 Safety updates

During the active[1]/enrollment phase of the study, safety updates will be provided via e-mail to the different members on a monthly basis for each patient enrolled in the study.

These updates will be sent by e-mail and include data specified in ATTACHMENT 2 - DSMB MONTHLY updates. If requested by the DSMB members, the SAE report and relevant source data (if available) will be provided.

For the remaining duration of the study, monthly updates will be provided during the data collection period.

The DSMB will review the data and provide their feedback via email to IQVIA MedTech.

A written statement must be prepared by each DSMB member within 10 calendar days of receipt of the safety update. This statement will involve the following 2 options:

1. No safety concerns
2. Safety concern.

In case of a safety concern, a rationale should be given. In case no answer is received from 1 or more of the members and/or no 2 equal responses are received from at least 2 members, an urgent reminder to the members that did not yet responded is sent by IQVIA MedTech.

In case the DSMB considers that there might be a safety issue, Polyganics will be informed and a DMSB meeting will be set up as soon as possible.

[1] *The active phase is considered as the period of enrollment up to the moment of the follow up related to the primary endpoint of the Study. In case the primary endpoint moment takes place during a long-term follow up (> 6 months after the previous visit), regular updates should be provided on a monthly basis during the moment of the data collection.*

4.4 Stopping rules

No termination criteria have been defined but may emerge based on case-by-case review of safety and outcomes.

4.5 DSMB Recommendations

Following each meeting, a formal report, separate from the minutes of the open and closed sessions, describing the DSMB recommendations and rationale will be prepared by IQVIA MedTech facilitator and sent to the members of the DSMB within 1 week after the meeting. The report will divulge no detail of DSMB discussions, only the final recommendation (ATTACHMENT 3 - DSMB MEETING REPORT).

Once approved by all members, the report will be sent to Polyganics.

The DSMB may recommend suspension or termination of the study based on the detection of unanticipated safety issues, such as higher event rates than anticipated, composite and/or individual major adverse events, primary endpoints, device failures, or unexpected/unanticipated SAEs and which might indicate there is a safety concern for the subject population, users or others. The DSMB will take into account the incidence and nature of the reported events, the causal/temporal relationship of the events to the device, previous experience with the device, and the known event rate from the literature. In the event of recommendation for suspension or termination, the DSMB Chairperson will promptly notify IQVIA MedTech facilitator. IQVIA MedTech will arrange a meeting or teleconference with Polyganics, IQVIA MedTech, and the DSMB to occur (if possible) within 5 working days.

While DSMB recommendations are not legally binding, they do require professional consideration by Polyganics.

4.6 Response to DSMB Recommendations

If the DSMB recommends continuation of the study without modification, no formal response will be required. However, if the recommendations request action, such as a recommendation for termination of the study or modification of the protocol, the DSMB will request that Polyganics provide a formal written response, within 5 working days, stating whether the recommendations will be followed and the plan for addressing the issues.

Upon receipt, the DSMB will consider the response of Polyganics and will attempt to resolve relevant issues, resulting in a final recommendation. Appropriate caution will be necessary during this process to avoid compromising study integrity or the ability of Polyganics to manage the study, should the study continue. Polyganics will agree to disseminate the final decision to the appropriate regulatory agency, EC/IRBs, and investigators within an appropriate time.

In the unlikely event of irreconcilable differences between the DSMB and Polyganics, especially regarding study termination or other substantial study modifications, the DSMB can, based on ethical considerations, step down as advisory board and express objection to continue monitoring the current study. This decision will be communicated to Polyganics and IQVIA MedTech.

Public disclosure of the final decision of Polyganics or DSMB recommendations will be at the discretion of Polyganics. Neither the DSMB nor IQVIA MedTech will make any public announcements either as a group or individually.

5 AMENDMENTS TO THE DSMB CHARTER

This DSMB charter can be amended as needed during the course of the study. Information to be included as amendments will be any modifications or supplements to the reports prepared for the DSMB, as well as amendments to other information addressed in this charter. Each revision will be reviewed and agreed upon by Polyganics, IQVIA MedTech, and the DSMB. All versions of the charter will be archived in accordance with this document.

6 ARCHIVING OF DSMB ACTIVITIES AND RELATED DOCUMENTS

All DSMB documentation and records will be retained by IQVIA MedTech until final study transfer to Polyganics. Access to archived data will be controlled by the IQVIA MedTech which will release the information only as specified in this charter or as required by law.

7 CONFIDENTIALITY

All materials, discussions, and proceedings of the DSMB are confidential. Members, and other participants in DSMB meetings, are expected to maintain the confidentiality.

ATTACHMENT 1 - CONTACT DETAILS

Data Safety and Monitoring Board

Name and Function:	Ruben Dammers<br>DSMB Chairperson
Address:	Dept. of Neurosurgery, NA-2112<br>Erasmus MC Stroke Center<br>PO Box 2040<br>3000 CA Rotterdam, the Netherlands
Phone:	+31-6-12055831
Fax:	
E-mail:	r.dammers@erasmusmc.nl

Name and Function:	Ricky Rasschaert<br>DSMB member
Address:	BVBA<br>Dr. Rasschaert Ricky – Dr. Verbeke Sofie<br>Boniverlei 28<br>2650 Edegem<br>Belgium
Phone:	Tel: +32 34940221<br>Mob: +32 476 799 208
Fax:	
E-mail:	ricky@dr-rasschaert.be

Name and Function:	William Peter Vandertop<br>DSMB member
Address:	VUMC | 2F-020 | De Boelelaan 1117, 1007 MB Amsterdam<br>AMC | H2-242 | Meibergdreef 9, 1105 AZ Amsterdam, the Netherlands
Phone:	Tel: +31-20 – 444 3725 | 06 – 2547 0560 (VUMC)<br>Tel: +31-20 – 566 3316 | #23545 (AMC)
Fax:	
E-mail:	wp.vandertop@amsterdamumc.nl

ATTACHMENT 2 - DSMB MONTHLY UPDATES

In case of regular (monthly – depending on the phase of the study as described above) updates, the following information will be provided to all DSMB members:

Safety

- Overview of the reported event(s)[2] including the following information:
    - Unique subject identifier.
    - Treatment arm
    - Date of event including calculation of days since procedure.
    - Type/ description of event.
    - SAE (Yes/No)
    - Relation to the study device
    - Relation to procedure
    - Event action / treatment and outcome.
    - Device deficiencies

Study conduct

- Current enrolment status (only during enrollment phase)
- Protocol deviations
- Subject follow- up status (overview of follow up visits completed to date)

[2] Tabular overview in PDF and xlsx formt

ATTACHMENT 3 - DSMB MEETING REPORT

To: Polyganics
Meeting date: DD-MMM-YYYY
Study: ENCASE II
Meeting Attendees: <...>

The DSMB charged with the review of safety data for ENCASE II study reviewed DSMB Data Report dated DD-MMM-YYYY

Summary of discussions in open session of the meeting: <...>

As a result, the DSMB recommendation is:

☐ To continue the study unmodified until next scheduled meeting.

☐ To continue the study unmodified, and plan an additional meeting: DD-MMM-YYYY (to be confirmed with Sponsor)

☐ To continue the study unmodified and request additional expert review/analyses.

<Describe and provide timelines of additional review>

☐ To set up a meeting with Polyganics to discuss concerns of safety within the ENCASE II study as outlined below.

☐ To suspend the study due to <...>

☐ To terminate the trial for the reasons outlined below.

Additional Comments:

<NAME>

Chairperson, Data Safety Monitoring Board for ENCASE II

Signature: Date:
